# Inhibitory effect of gene combination in a mouse model of colon cancer with liver metastasis

**DOI:** 10.3892/etm.2014.1809

**Published:** 2014-06-25

**Authors:** TONG DU, HONGXIN NIU

**Affiliations:** Department of Psychology, Affiliated Hospital of Shandong Academy of Medical Sciences, Jinan, Shandong 250031, P.R. China

**Keywords:** colon cancer, liver metastasis, nude mouse model, gene therapy

## Abstract

The aim of the present study was to establish an animal liver metastasis model with human colon cancer and investigate the inhibitory effect of the wild type (WT) p53 gene combined with thymidine kinase/ganciclovir (TK/GCV) and cytosine deaminase/5-fluorocytosine (CD/5-FC) systems on liver metastasis of colon cancer. A nude mouse liver metastasis model with human colon cancer was established via a spleen cultivation method. A total of 32 nude mice were randomly divided into four groups, each group with eight mice. Group 1 mice received splenic injections of SW480 cells (control group), while group 2 mice were injected with SW480/p53 cells in the spleen. Group 3 mice were administered splenic injections of SW480/TK-CD cells, and GCV and 5-FC were injected into the abdominal cavity. Finally, group 4 mice received splenic injections of SW480/p53 cells mixed in equal proportion with SW480/TK-CD cells, as well as GCV and 5-FC injections in the abdominal cavity. These cells described were constructed in our laboratory and other laboratories. The number of liver metastatic tumors, the liver metastasis rate, conventional pathology, electron microscopy and other indicators in the nude mice of each group were compared and observed. The nude mouse liver metastasis model with human colon cancer was successfully established; the liver metastasis rate of the control group was 100%. The results demonstrated that the rate of liver metastasis in the nude mice in each treatment group decreased, as well as the average number of liver metastatic tumors. Furthermore, the effect of the treatment group with genetic combination (group 4) was the most effective, demonstrating that WTp53 had a synergistic effect with TK/GCV and CD/5-FC. Therefore, the present study successfully established a mouse model of liver metastasis with colon cancer by injecting human colon cancer cells in the spleen. Combined gene therapy was shown to have a synergistic effect, which effectively inhibited the formation of liver metastasis from colon cancer.

## Introduction

In recent years, the incidence of colon cancer has shown a gradual upward trend in China, becoming the third most common tumor type endangering human health (1,2). Liver metastasis from colon cancer is extremely common and is the main reason for clinical treatment failure, which has subsequently impacted long-term survival and prognosis (3–5). As a result, the prevention and treatment of colon cancer with liver metastasis is a clinically important topic that requires further investigation. At present, surgical resection is the preferred method of treatment for colon cancer with liver metastasis, while other conventional treatment methods include chemotherapy, radiation, radio frequency therapy, freezing, microwaving or using a laser, percutaneous ethanol injection and radioactive seed implantation. However, gene therapy and immunotherapy techniques performed in recent years have become highly promising strategies for colon cancer treatment (6,7). The establishment of animal models of colon cancer with radical resection of liver metastasis has improved the investigations into the mechanisms underlying liver metastasis in human colon cancer; thus, improving the development of novel, effective liver metastasis prevention and treatment programs. Previously, an SW480 nude mouse subcutaneous transplanted tumor model was established and the wild type (WT) p53 gene was combined with thymidine kinase/ganciclovir (TK/GCV) and cytosine deaminase/5-fluorocytosine (CD/5-FC) systems, which were shown to significantly inhibit the growth of subcutaneous transplanted tumors and prolong the survival of mice (8). However, whether this method can affect liver metastasis in colon cancer remains unclear. On the basis of the aforementioned evidence, the present study successfully established an SW480 nude mouse liver metastasis model and further observed the role of combined gene therapy in the prevention and treatment of liver metastatic tumors. The aim of the present study was to provide a novel approach to clinically prevent colon cancer radical resection of liver metastasis and treat liver metastatic tumors of colon cancer.

## Materials and methods

### Collection and preparation of the cell suspension

A human colon cancer cell line, SW480, was purchased from Shanghai Institute of Biological Cells (Shanghai, China). These cells have point mutations in the eighth and ninth exons of the p53 gene. SW480/p53 and SW480/TK-CD were built in an *in vitro* experiment and were shown to stably transfect p53 and TK and CD double suicide genes, respectively (8). Human colon cancer SW480 cells, SW480/p53 cells and SW480/TK-CD cells were cultured and passaged in RPMI-1640 medium (Hyclone, Logan, UT, USA) containing newborn calf serum (Hyclone) and incubated at 37°C constant temperature to obtain a sufficient number of cells. Next, the well-grown cells of ~80% confluence (Hyclone, Logan, UT, USA) were collected to establish a cell suspension at a concentration of 5×10^7^, which were then inoculated to the spleen on an ice bath.

### Grouping

A total of 32 BALB/c female nude mice (age, 4–6 weeks; weight, 14–20 g) were purchased from the Experimental Animal Center of Shandong University (Jinan, China) and reared under specific-pathogen free conditions. The study was conducted in strict accordance with the recommendations in the Guide for the Care and Use of Laboratory Animals of the National Institutes of Health, and the animal use protocol was reviewed and approved by the Institutional Animal Care and Use Committee of the Affiliated Hospital of Shandong Academy of Medical Science (Jinan, China). A total of 32 nude mice were randomly divided into four groups (n=8 per group). Group 1 mice received splenic injections of SW480 cells (control group), while group 2 mice were injected with SW480/p53 cells in the spleen. Group 3 mice were administered splenic injections of SW480/TK-CD cells, and GCV (Roche, Basel, Switzerland) and 5-FC were injected into the abdominal cavity. Finally, group 4 mice received splenic injections of SW480/p53 cells mixed in equal proportion with SW480/TK-CD cells, as well as GCV and 5-FC injections (Sigma, San Francisco, CA, USA) in the abdominal cavity (9).

### Establishment of a liver metastasis animal model with colon cancer

An injection method was used to establish the model, which firstly involved weighing the nude mice and injecting 45 mg/kg sodium pentobarbital (1%) into the abdominal cavity for anesthesia. Following forced entry, the skin in the surgical field was disinfected and a 0.5–1.0-cm back left oblique incision was made (below the junction of the left armpit bottom line and costal margin) into the abdomen to expose the spleen, which was then raised out of the abdominal cavity gently. The SW480, SW480/p53 and SW480/TK-CD colon cancer cells were then slowing injected, according to the group, into the spleen of the nude mice using a fifth mode of a needle. Each nude mouse was injected with 0.2 ml cell suspension (1×10^7^/per) for at least 3 min, following which the spleen capsule was found to swell and grow white. Following the injection, it was a requirement that the needle eye was oppressed with a 75% alcohol swab for 2 min to stop the bleeding and kill the cancer cells, which may exosmose in order to prevent metastasis in the abdominal cavity. Next, the spleen was placed in the original position and the abdomen was closed. The principles of aseptic surgery were followed during the whole process.

### Suicide gene prodrug therapy

One day after the spleen inoculation, normal saline (the same volume as suicide gene prodrugs) was injected into the abdominal cavity of the mice in groups 1 and 2, while the suicide gene prodrugs, GCV and 5-FC, were injected into the abdominal cavity of the mice in groups 3 and 4 for ten consecutive days; the injection doses were 100 mg/(kg/d) GCV and 500 mg/(kg/d) 5-FC.

### Efficacy observations

Nude mice in each group were autopsied six weeks following surgery to observe whether metastasis of the liver and other areas had occurred. The livers were removed and fixed with 10% formalin solution for one night. Next, the number of metastatic tumors (metastatic nodules on the liver surface that were visually counted), liver metastasis rate, conventional pathology, electron microscopy and other indicators were monitored.

### Statistical analysis

SPSS 16.0 (SPSS, Inc., Chicago, IL, USA) software was used for statistical analysis. The χ^2^-test and Student’s t-test were used to compare the liver metastasis rate and average number of liver metastatic tumors, respectively. P<0.05 was considered to indicate a statistically significant difference.

## Results

### Metastasis cases of the liver and other areas in each group

**Figure 1:**
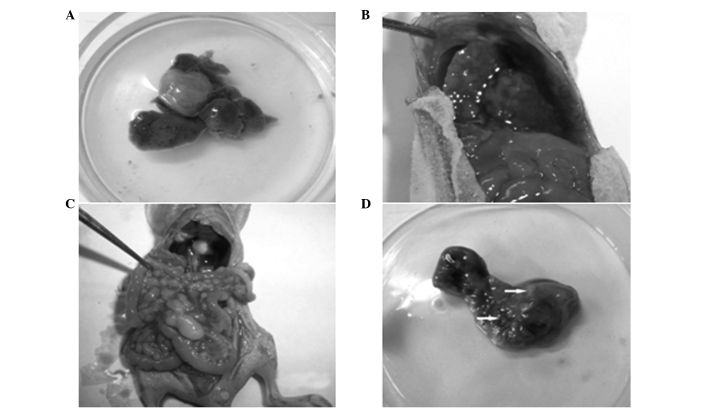
Images showing liver metastatic tumors with (A) isolating large nodules and (B) diffuse miliary nodules, (C) abdominal cavity metastatic tumors (mesentery and omentum) and (D) spleen *in situ* tumors.

Grey nodules of varying numbers were present on the liver surface of nude mice with liver metastasis; as shown in Fig. 1, a large isolating nodule with a maximum diameter of 3 cm (Fig. 1A) and multiple miliary nodules (Fig. 1B) were observed. The liver volume decreased and the texture was brittle and hard, with ulceration observed in areas with large nodules. A normal liver is usually bright red with a soft texture.

The liver metastasis rate and metastatic tumor numbers of each group are shown in Table I. Comparisons between each treatment group and the control for the average number of liver metastatic tumors were statistically significant (P<0.01). With regard to the liver metastasis rate, no statistically significant difference was observed when comparing groups 2 and 1 (P>0.05). However, when comparing groups 3 and 4 with group 1, statistically significant differences were observed (P=0.013 and P=0.001, respectively), indicating that combined gene therapy significantly reduced the number of liver metastatic tumors from colon cancer and reduced the incidence of liver metastasis.

With regard to metastasis to other organs, one or more cases were observed in the lymph nodes, diaphragm and abdominal cavity in the nude mice of the control group (Fig. 1C); however, metastasis was not observed in the heart, lung, brain or kidney. Metastasis was not present in other organs of the nude mice in the treatment groups. The mice with liver metastasis all had *in situ* implanted tumor formation in the spleen (Fig. 1D; all metastasis cases were confirmed by pathological examination from the same adenocarcinoma).

### Light microscopy observations

**Figure 2:**
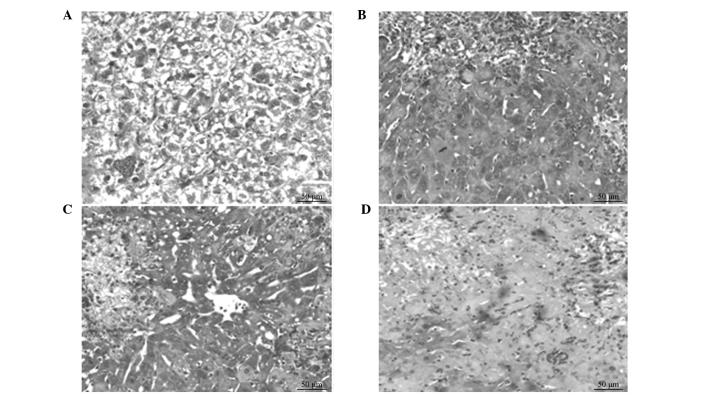
Pathological sections of nude mouse liver metastatic tumor tissues in groups (A) 1, (B) 2, (C) 3 and (D) 4 (hematoxylin and eosin stain; magnification, ×200).

Tumor cell growth in the control group was active and a lobular structure remained distinguishable. The tumor cells were significantly heteromorphic, with a round nucleus that was placed in the middle of the cell. The nuclei were large and deeply stained, and nucleus division increased while the amount of cytoplasm was lower. Fibrosis and inflammatory cell infiltration were not observed, with apoptosis present only in small areas (Fig. 2A). Necrotic tissue was present in the treatment groups and apoptotic cells were observed, with cell shrinkage and cytoplasm and nuclear condensation. Apoptotic bodies were observed following nuclear fragmentation and dissolution. In groups 2 and 3, the tumor tissues of the nude mice exhibited a small area of tumor necrosis (Fig. 2B and C); however, necrosis was observed in the majority of the regions in group 4. Tumor cell structures were unclear and inflammatory cells were observed in the necrotic tissues (Fig. 2D).

### Electron microscopy observations

**Figure 3:**
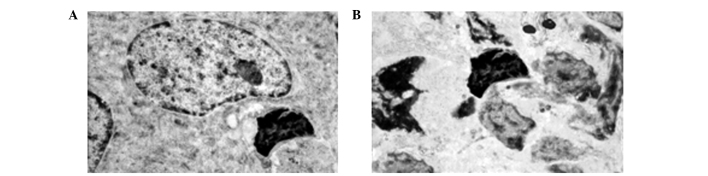
Electron microscopy images showing the ultrastructure of liver metastatic tumor tissues in the (A) control and (B) treatment group 4 (magnification, ×6,000).

As demonstrated in Fig. 3A, tumor cells in the control group were irregular with a large volume and large nucleus. Nucleoli were prominent, nucleus division was easy to observe and there were a high number of organelles in the cytoplasm. A large number of tumor cells in the treatment groups were soma-condensed, with cytoplasmic concentration, ribosomes and mitochondria aggregation, nuclear condensation and nuclear fragmentation. The complete pericellular membrane of the apoptotic bodies was also observed (Fig. 3B).

## Discussion

Spleen planting and cecal wall planting methods are most commonly used to establish an experimental liver metastasis model with colon cancer. Other routes are possible, including implantation within the portal vein, ileocolic vein and liver, however, these are rarely used due to operational difficulties (10,11). When using the cecal wall planting method, the range of tumor metastases is wide, with a relatively low incidence and slow development of liver metastasis. With regard to the spleen planting method, the incidence of liver metastasis may be as high as 100%, extrahepatic metastasis rarely occurs and the development of liver metastasis is rapid (12,13). The establishment of this model aims to study liver metastasis of colon cancer; thus, the spleen injection route was selected in the present study. Although concurrent spleen *in situ* tumors were present, the spleen immune function and the inherent host antitumor immunity were retained, and were more consistent with the complex environment of tumor occurrence and development in the human body.

The ability to maintain the original biological characteristics of tumor cells is key to animal model establishment. The present study simulated the process of cancer cells backflowing into the portal vein and liver, which resulted in hematogenous dissemination and liver metastasis following the resection of colon cancer; thus, an SW480 nude mouse liver metastasis model was successfully constructed. This model did not affect the invasion and metastasis biological characteristics of human colon cancer cells. Pathological sections in the control group demonstrated complete consistency with regard to the histological structure of the liver metastatic tumors with human colon adenocarcinoma.

In the present study, a number of elements of the model establishment were noted for future reference. Firstly, the biological characteristics of the original tumor cells *in vitro* cultivation and preparation of suspensions should be maintained. Following enzyme digestion and treatment, certain components in the surface of tumor cells changed, which may affect the growth and metastasis potential of tumor cells. Therefore, the enzyme digestion time should be well controlled, the cells should be gently pipetted and the centrifugation speed should not exceed 1,200 g. Secondly, an incision, 0.5–1.0 cm in length, should be cut in the middle back to minimize the trauma and reveal the spleen under easy and secure surgery. Thirdly, during the injection, the under part of the spleen should be pulled from the incision and the needle should be inserted horizontally along the spleen axis with a depth of ≥0.5 cm in order to prevent the spleen from being easily punctured and suspensions from overflowing from the injection site. Fourthly, the number of injection cells should be large enough to reach 1×10^7^ in each mouse, as the implanted tumor cells must escape attack from numerous natural killer cells and activated phagocytic cells, which secrete tumor necrosis factors, in order to cause vascular invasion and liver metastasis. Finally, the injection time should be at least 3 min in order to avoid splenic capsule rupture due to excessive tension.

Currently, combined gene therapy is emerging as a popular tumor therapeutic strategy. p53 gene mutations are the most common genetic alterations in colon cancer (14), and tumor cells with p53 mutations can compete with the killing effect of suicide gene transformation prodrugs on tumor cells; thus, affecting the therapeutic effect of the suicide gene (15–18). In a number of suicide gene systems, studies on the TK and CD genes have been highly detailed, with definite effects observed. The TK and CD double suicide gene alliance has been demonstrated to be more efficient, more broad-spectrum, more secure, less simple to produce drug resistance and able to reduce the doses of prodrugs (18–20). The most important feature of suicide gene therapy is the bystander effect, which overcomes the disadvantages of low gene transduction and significantly expands the killing effect of suicide genes. Numerous studies have demonstrated that double suicide gene systems, as compared with a single suicide gene, may produce a more powerful bystander effect (21–23). In addition, the bystander effect may expand the therapeutic effect of the WTp53 gene in tumors into adjacent cells and consequently produce an enhanced tumor suppressive effect (24,25).

The application of gene therapy firstly demands the transfected gene to exhibit certain expression efficiency. In addition, the spliced gene should be limited among the target cells in order to maximize the killing of tumor cells without damaging normal cells. Therefore, improving the targeted gene transfer techniques and the efficiency of gene targeting transfection is key to current gene therapy. In the present study, prebuilt SW480/p53 and SW480/TK-CD cells containing the target genes were transplanted into the spleen to produce liver metastasis, and the problem of gene targeting transduction was successfully resolved (25). Following the transplantation of cancer cells into the spleen, the prodrugs, GCV and 5-FC, were timely administered on the portal vein system into the abdominal cavity. This administration route allowed the abdominal cavity, portal vein and liver to be subjected to a constant, high concentration of the drugs. The selected route and timings of the aforementioned genes and prodrugs were the most efficient combination to produce a more direct, targeted and specific gene therapy effect. The introduction of the WTp53 gene not only incorporates its normal tumor suppressor function to compete with the mutated p53, but also enhances the performance of the double suicide genes and demonstrates a more powerful bystander effect. The results demonstrated that the combination of the WTp53 gene with the TK/GCV and CD/5-FC systems exhibited a synergistic effect. In addition, compared with single gene application, this therapy markedly reduced the incidence of colon cancer liver metastasis and the number of liver metastatic tumors. Therefore, the present study has provided a novel approach for the prevention of liver metastasis following radical resection of colon cancer and for the treatment of colon cancer liver metastatic tumors.

The role of combined gene therapy is not a simple composition, but an organic combination, the complementary and synergistic effects of which may markedly improve the tumoricidal effect (26). However, *in vivo* animal experiments differ from humans. In reality, it is not possible to transfect the target genes into tumor cells and then transform the tumor cells containing the target genes into tumors, as has been performed in the present study. Thus, the target genes are unable to be transfected into each tumor cell. Therefore, future studies are required to design efficiently targeted gene delivery vectors that are able to achieve safe and controllable gene expression with more gene combinations.

## Figures and Tables

**Table I:** Effect of combined gene therapy on the average number of liver metastatic tumors and the liver metastasis rate.

Group (n=8)	Liver metastatic tumors, n	Liver metastasis rate, %
1	12.13±6.20	100
2	3.13±2.23	87.5
3	0.50±0.76	37.5
4	0.13±0.35	12.5
